# Complications of Microvascular Upper Lip and Free Grafted Nasal and Eyebrow Replantation After Assault via Human Bite

**DOI:** 10.7759/cureus.4631

**Published:** 2019-05-10

**Authors:** Garrison A Leach, Jaclyn N Lundberg, Travis C Holcombe

**Affiliations:** 1 Surgery, Creighton University School of Medicine, Phoenix, USA; 2 Plastic Surgery, Creighton University School of Medicine, Phoenix, USA

**Keywords:** tpa, amputation, plastic surgery, microsurgery, trauma, myocardial infarction, stroke

## Abstract

Amputation of facial soft tissue, particularly avulsion due to human bite, is an uncommon injury that has severe cosmetic and functional implications. Microsurgical replantation has the potential for superior aesthetic outcomes and restoration of function. We report a case of a 46‐-year-old male who sustained avulsion injuries from human bites, which included portions of his eyebrow, nose, and upper lip. Artery and vein microvascular replantation was performed on the upper lip. The amputated eyebrow and nasal segments were replanted in a similar fashion to a skin graft. On post-operation day 1, our patient suffered an ischemic stroke followed by a myocardial infarction requiring systemic tissue plasminogen activator (tPA) treatment. Following administration of tPA, there was continuous bloody discharge from the replant sites and the eyebrow, nose, and upper lip began to appear increasingly dusky. Our patient was determined to be a high-risk candidate for immediate revision surgery and he subsequently underwent a six-stage secondary reconstruction. At his most recent four-month follow-up, our patient is satisfied with his cosmetic and functional outcomes. This was a case of failed microvascular upper lip replantation and eyebrow and nasal replacement complicated by stroke and myocardial infarction. The authors review the common complications in replantation, particularly pertaining to upper lip reanastamosis, and discuss a potential novel complication encountered in this case relevant to both free graft and microvascular replantation.

## Introduction

Amputation of facial soft tissue, particularly in the context of avulsion due to human bite, is an uncommon injury that has severe cosmetic and functional implications. In the specific case of lip amputation, microsurgical replantation has the potential for superior aesthetic outcomes as well as restoration of sensory and motor function. Since James reported successful microsurgical replantation of the lip in 1976, there have only been 21 cases reported, four of which were due to human bites [1-6]. Due to the paucity of reports on this, complications are likely to be underreported. We present a case of extensive human bite injuries, resulting in amputated eyebrow and nasal segments replanted similarly to skin graft and upper lip amputation repaired with microvascular replantation; recovery was subsequently complicated by myocardial infarction and ischemic stroke and secondary reconstruction were required.

## Case presentation

**Figure 1:**
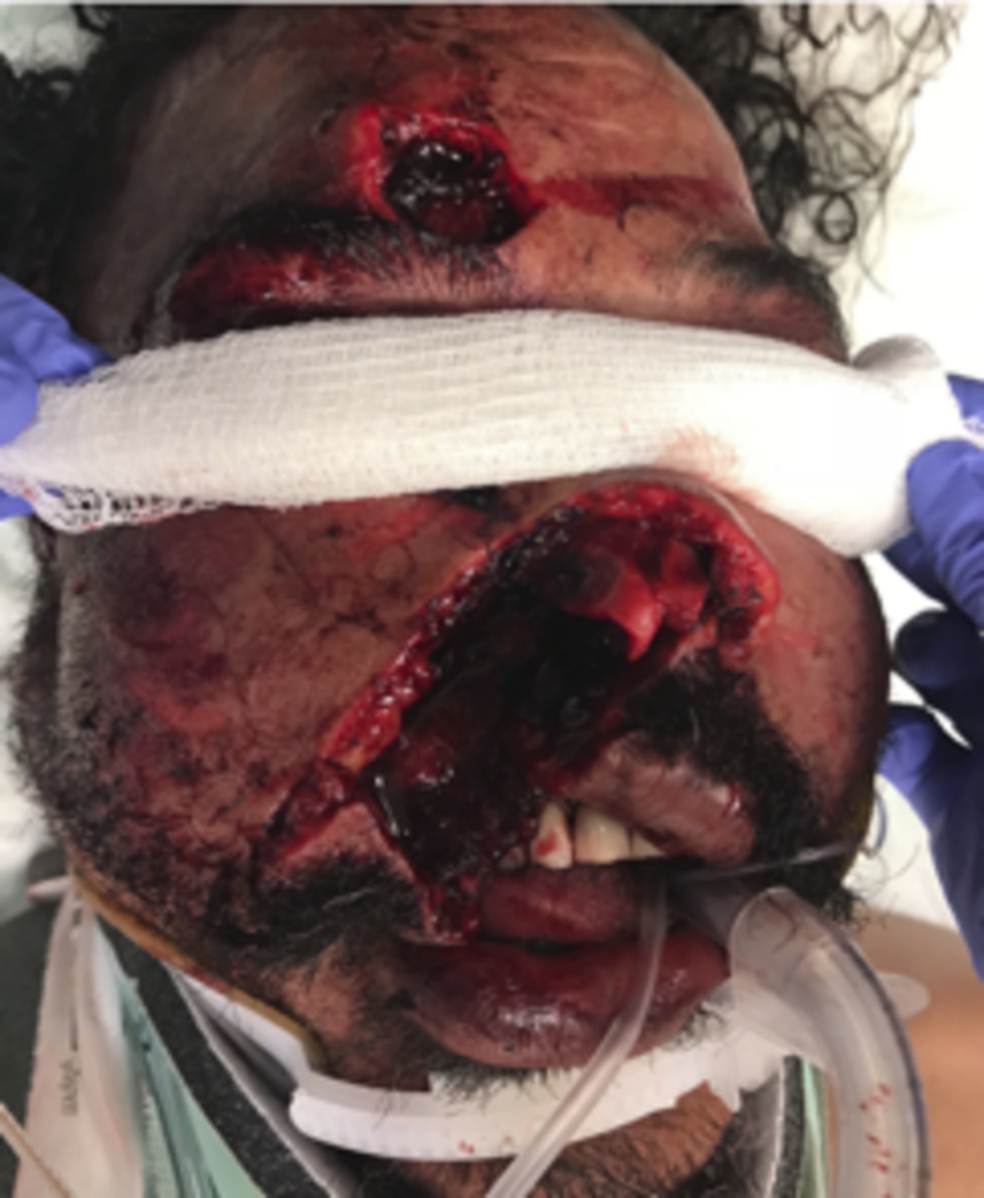
Patient upon presentation to the trauma bay

A 46-year-old male with a past medical history of hypertension presented to our trauma bay with complex injuries of the right nose and midface, which included an amputated nose, upper lip and right eyebrow approximately 40 minutes after an assault including numerous human bite wounds. His right ala, right sidewall, and right upper lip were entirely missing, with nasal airways on each side visible. The amputated specimens included nasal tissue which measured approximately 4.0 x 4.0 cm, the eyebrow 2.0 x 2.0 cm, and the upper lip 1.0 x 11.7 cm (Figure 1). After rapid sequence intubation, assessment, and stabilization, the patient was taken to the operating room about one hour after the initial presentation for reattachment and wound reconstruction.

In the OR, the superior labial artery and a small outflow vein, which was likely part of the superior labial venous plexus, were identified and isolated from the lateral portion of the lip. However, the nasal segment contained no identifiable viable artery or vein. Therefore, the nasal and eyebrow portions were replanted similarly to full thickness skin grafts and the upper lip was replanted using microvascular techniques. The segments of each vessel were flushed with heparinized saline. Then, the superior labial artery was anastomosed using 9-0 nylon interrupted sutures, followed by anastomosis of the vein using 9-0 nylon. After three hours and 19 minutes of operating time, arterial blood flow was immediately apparent, but venous flow was not definitive. The wounds of the upper eyelid and right cheek were debrided and then closed.

**Figure 2:**
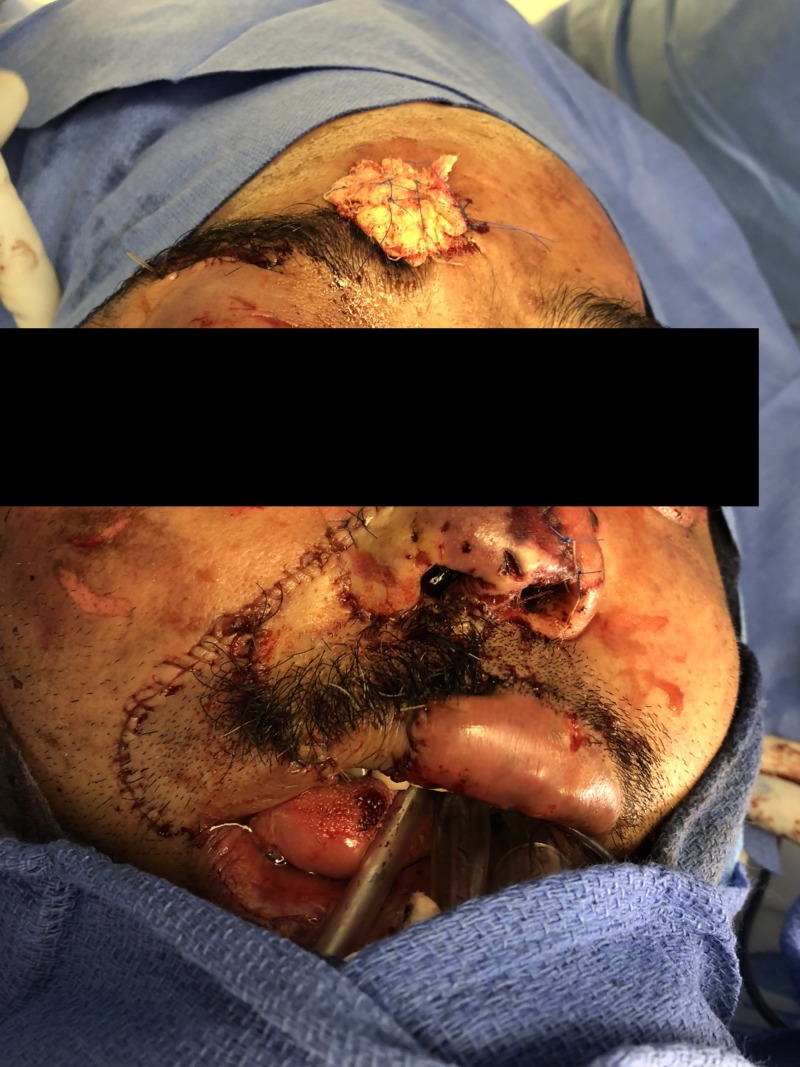
Patient after initial replant

To improve the chance of tissue survival, our patient was scheduled to receive hyperbaric oxygen treatment within 24 hours post-operation. However, the patient reported that his ears could not tolerate the pressure and therapy was postponed until otolaryngology could insert tubes at the bedside. Additionally, he was receiving 30 mg of enoxaparin daily to mitigate the chance of outflow venous thrombosis. The replaced nasal and eyebrow tissue and the microsurgically replanted upper lip appeared to have some minor ischemia at the lateral margins (particularly of the nasal tip) that would most likely require revision, but the majority of the three portions appeared healthy (Figure 2).

Later that day, the patient reported upper extremity numbness. After a non-contrast CT showed no intracranial abnormality, he received 8.34 mg bolus of tPA followed by 75.1 mg of IV tPA over the course of an hour for possible ischemic stroke. Subsequent MRI showed a small acute left frontal cortical infarction. The next morning the patient reported chest discomfort and an EKG showed ST-segment elevation of inferior leads with elevated troponin peak of 132.2 due to myocardial infarction. The patient then underwent left heart catheterization that showed adequate flow and no need for further intervention. After these events, he received 30 mg of enoxaparin twice a day per protocol. 

**Figure 3:**
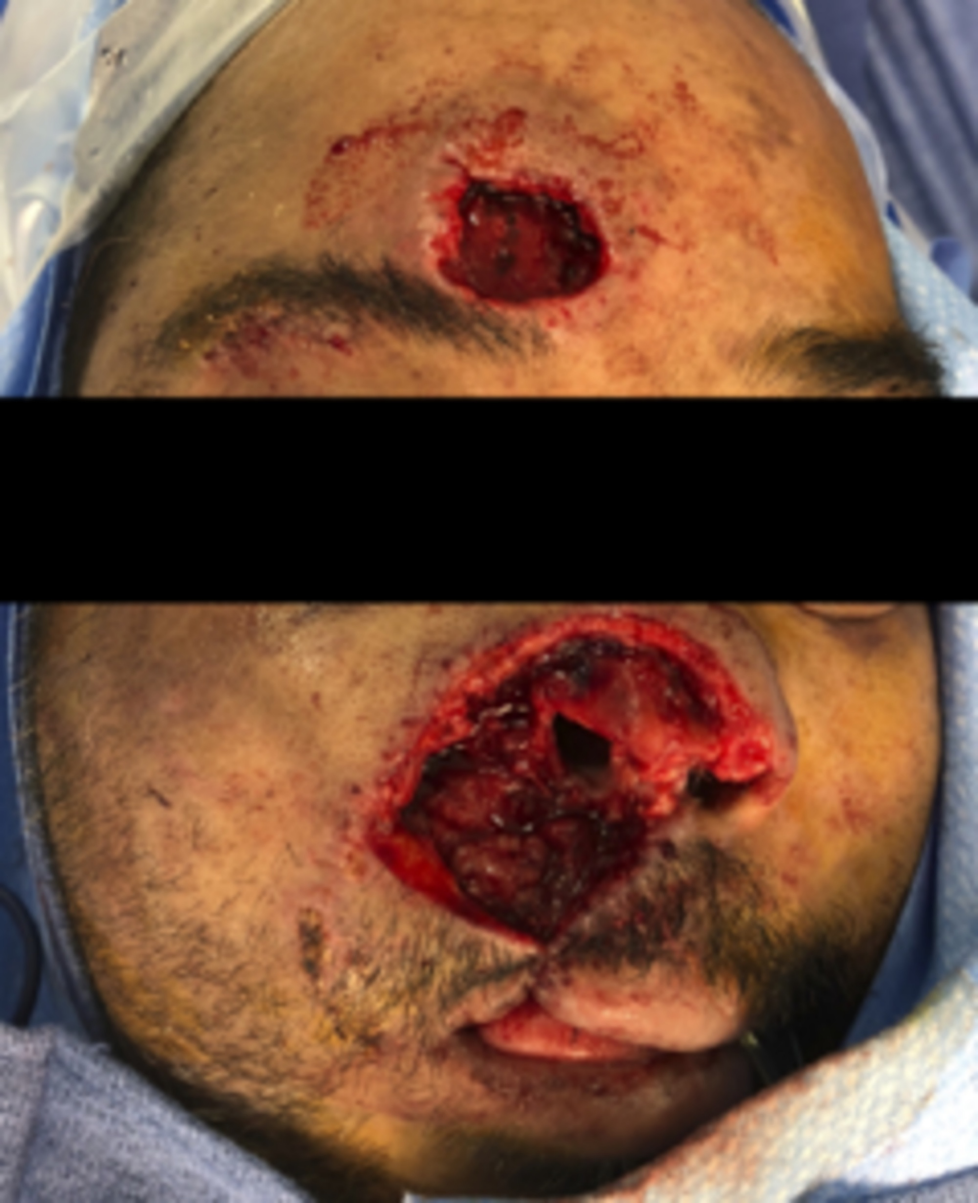
Patient post-debridement of necrotic replants

**Figure 4:**
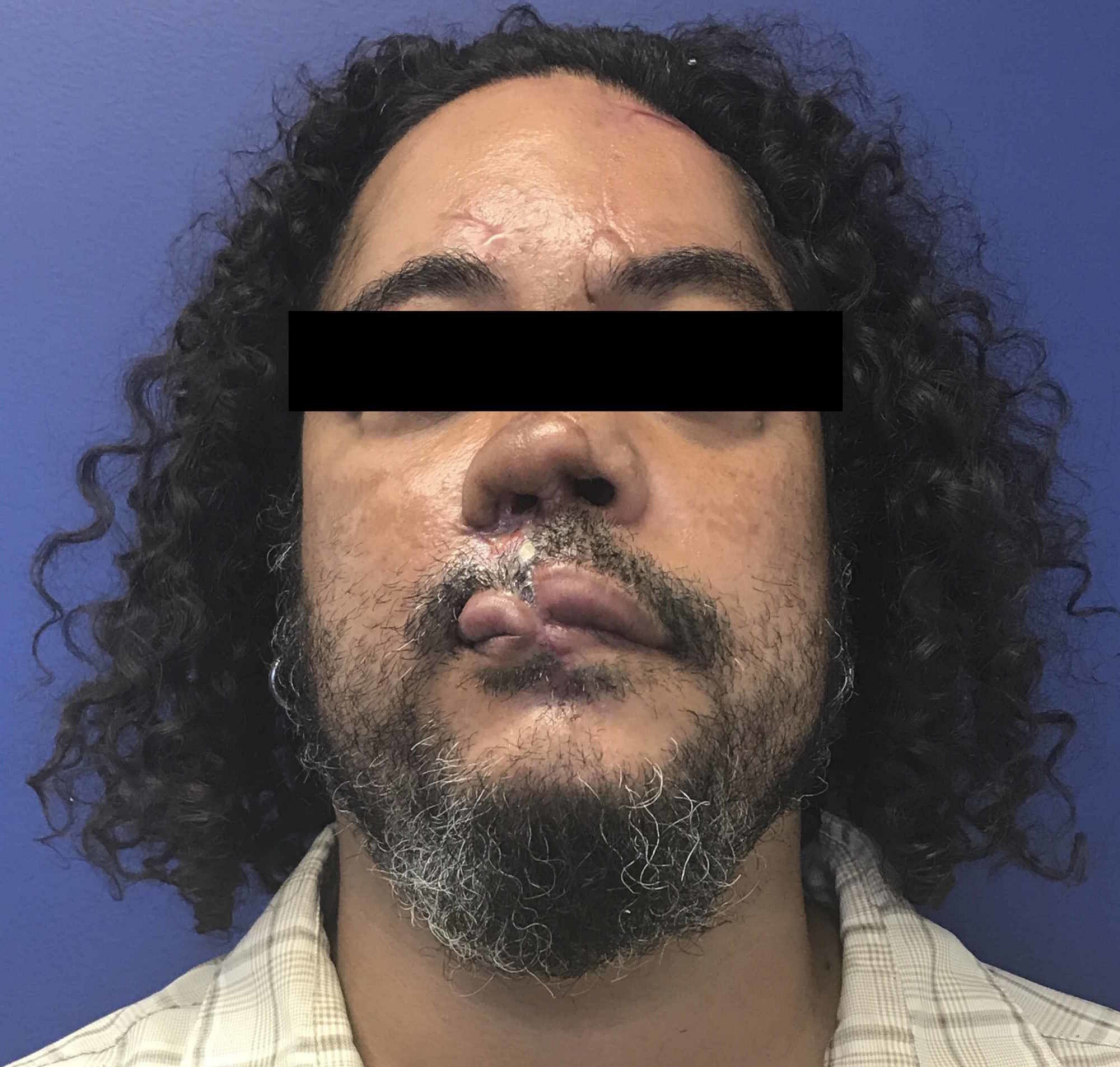
Patient after the second stage of paramedian flap, ear cartilage graft to nose, and full-thickness skin graft of the upper lip, but prior to Abbe flap

**Figure 5:**
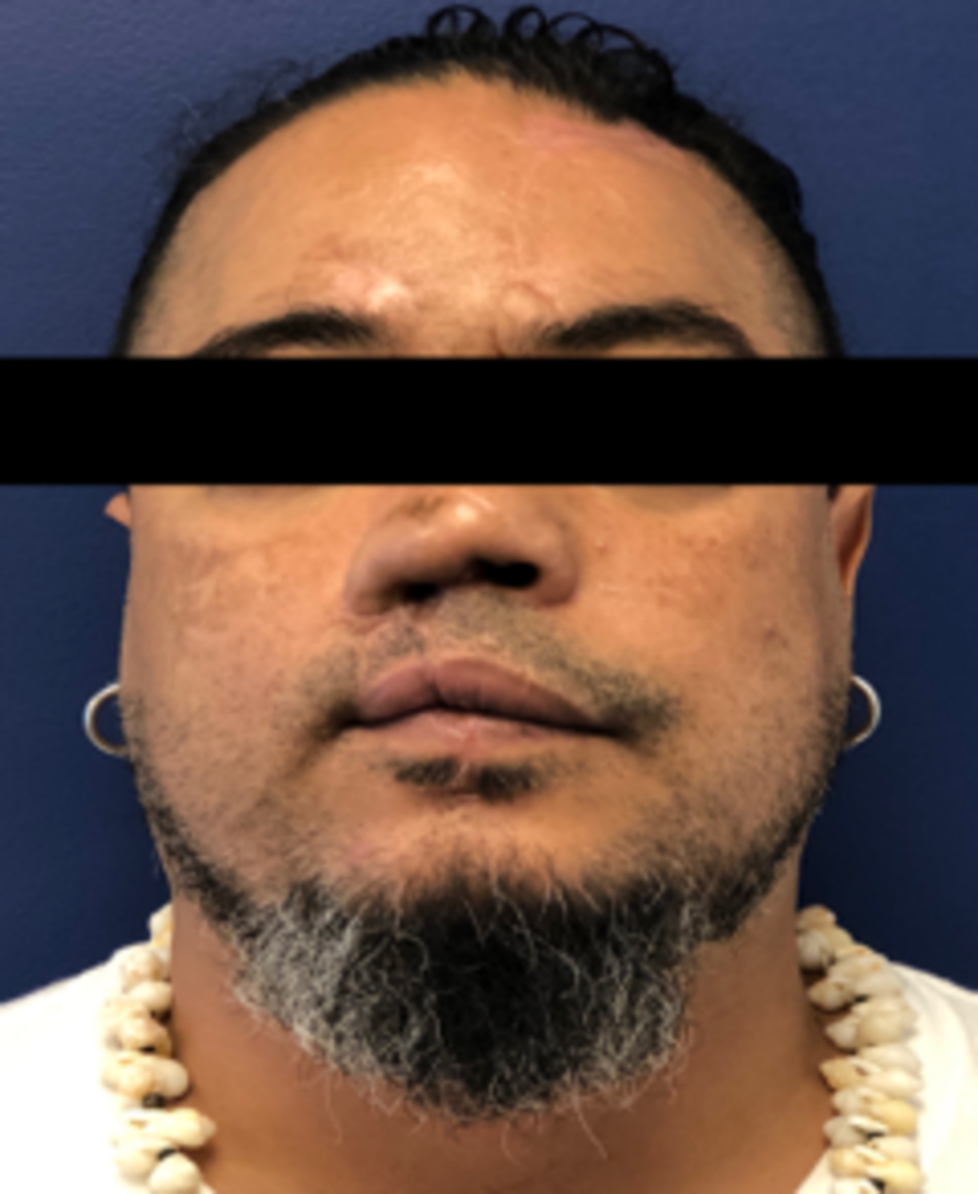
Patient four months after final revision surgery

After tPA treatment, there was marked continuous sanguineous discharge from the replant sites and the eyebrow, nose, and upper lip began to appear increasingly dusky. Our patient was determined to be a high-risk candidate for immediate revision surgery and plans for secondary reconstruction were made. The patient returned six days later for facial wound debridement of necrotic wounds of the upper lip, nose, and right forehead with subsequent placement of Integra artificial dermis (Figure 3). He subsequently underwent nose and upper lip advancement flaps to save oral competence. A month later, nasal reconstruction was undertaken with a left paramedian forehead flap and full-thickness skin graft to upper lip and nose. The second stage of reconstruction was done one month later including the second stage of the paramedian flap, ear cartilage graft to the nose, and full-thickness skin graft of the upper lip (Figure 4). Subsequent reconstruction one month later included an Abbe flap from the lower lip to the upper lip and final revision of the forehead flap. Overall, our patient was satisfied with the cosmetic and functional outcome of his reconstruction four months after his final surgery (Figure 5).

## Discussion

By presenting this case, the authors would like to highlight some of the common complications in replantation, particularly pertaining to upper lip reanastamosis. We also pose a new potential complication encountered in this case relevant to both free graft and microvascular replantation.

One complication of replantation specific to our patient is the anatomy of the upper lip in relation to bite injuries. Establishing venous anastomosis with an adequate flow in the upper lip is technically challenging because the superior labial vein is frequently a venous plexus [6-7]. Additionally, the crush and shear forces associated with a bite can result in extensive damage to the venous intima further complicating the establishment of good venous drainage [7-9]. Furthermore, maintaining outflow of venous drainage can also be difficult. To solve this problem, leeches have been employed to prevent venous congestion and anticoagulants have been implemented to prevent thrombus formation [8]. Unfortunately, we are unable to determine if thrombosis developed in our patient because immediate revision and exploration were subsequently cancelled due to the post-operative stroke and STEMI. Nonetheless, awareness of the challenge of achieving patent venous drainage is essential in management of microsurgical lip replants.

In addition, the administration of systemic tPA for stroke was a potentially unique complication that has not been described in replantation per the literature. As mentioned above, anticoagulants have frequently been used to improve venous drainage in microsurgery including replantation. Although at significantly smaller doses than those used for stroke, tPA has been administered both subcutaneously and via intra-arterial injection in free flaps to prevent venous obstruction [10]. However, we are not aware of any studies or reports on the effects of administration of a larger dose, systemic tPA in replantation. One well-known complication of tPA is post-surgical bleeding. Albeit different than replantation, post-operative bleeding is a noted factor for free flap failure [11]. It is not unreasonable to hypothesize that the same applies to replantation. Contrarily, post-operative bleeding from the replant site could also be indicative of venous thrombosis [11]. Grafts are particularly sensitive to ischemia as angiogenesis occurs the first week after surgery [12-13]. The fibrinolytic effects of tPA have altered neovascularization in animal models and as a result, it is possible a large dose, such as that in our patient, could compromise both microvascular replant and graft survival [14-15]. 

There were a number of other factors that we do not suspect to have played a role in our patients replant failure. We do not believe that ischemia time, delay in revascularization, or the duration of procedure contributed. It was estimated that it took 40 minutes from the injury to the initial presentation in the trauma bay and on arrival, the amputated tissues showed minimal signs of ischemia. Furthermore, considering the nature and extent of our patient's injuries in addition to the complexity of the procedure, the delay in getting him to the operating room as well as the actual procedure time were kept to a minimum. Metabolic status can also have a prominent effect on healing. Although his lipid panel demonstrated elevated triglycerides, our patient's albumin was within normal limits, and clinically, we had no concerns of poor metabolic states having a detrimental outcome on his course. Lastly, hypothermia was not a concern as our patient maintained a temperature of at least 37 degrees Celsius pre and post-op in addition to never dropping below 36 degrees in the operating room.

## Conclusions

We are unable to say for certain the exact mechanism of graft failure in this patient. Although there were several variables including the shearing and crush forces associated with bite injuries, the anatomy of the upper lip, and the technical difficulty of the procedure that all could have contributed to the demise of this patient's replants, the rapid and synchronous onset of ischemia following tPA in all three replants suggests that, although most likely not the sole causal element, tPA could be a contributing factor. Further research will need to be done to determine if administration of systemic tPA in tissue replantation contributes to free graft and replant failure.
